# Commiphora Extract Mixture Ameliorates Monosodium Iodoacetate-Induced Osteoarthritis

**DOI:** 10.3390/nu12051477

**Published:** 2020-05-19

**Authors:** Donghun Lee, Mi-Kyoung Ju, Hocheol Kim

**Affiliations:** 1 Department of Herbal Pharmacology, College of Korean Medicine, Gachon University, 1342 Seongnamdae-ro, Sujeong-gu, Seongnam 13120, Korea; dlee@gachon.ac.kr; 2 Korea Institute of Science and Technology for Eastern Medicine (KISTEM) NeuMed Inc., 88 Imun-ro, Dongdaemun-gu, Seoul 02440, Korea; jmg8586@neumed.co.kr; 3 Department of Herbal Pharmacology, College of Korean Medicine, Kyung Hee University, 26 Kyungheedae-ro, Dongdaemun-gu, Seoul 02447, Korea

**Keywords:** osteoarthritis, *Paeonia lactiflora*, Commiphora myrrha, analgesic, anti-inflammatory

## Abstract

Osteoarthritis (OA) is a chronic inflammatory joint disease that affects millions of elderly people around the world. The conventional treatments for OA consisting of nonsteroidal anti-inflammatory drugs and steroid have negative health consequences, such as gastrointestinal, renal, and cardiac diseases. This study has evaluated the Commiphora extract mixture (HT083) on OA progression as an alternative treatment in animal models. The root of *P. lactiflora* and the gum resin of *C. myrrha* have been in use as traditional medicines against many health problems including bone disorders since ancient time. The extracts of *P. lactiflora* root and *C. myrrha* gum resin were mixed as 3:1 for their optimal effects. Male Sprague-Dawley rats were injected with monosodium iodoacetate (MIA) into the knee joints to induce the symptoms identical to human OA. HT083 substantially prevented the loss of weight-bearing inflicted with MIA in rats. The MIA-induced cartilage erosion as well as the subchondral bone damage in the rats was also reversed. In addition, the increase of serum IL-1β concentration, a crucial pro-inflammatory cytokine involved in OA progression was countered by HT083. Furthermore, HT083 significantly reduced the acetic acid-induced writhing response in mice. In vitro, HT083 has shown potent anti-inflammatory activities by inhibiting the production of NO and suppressing the interleukin -1β, interleukin -6, cyclooxygenase-2, and inducible nitric oxide synthase expression in lipopolysaccharide -stimulated RAW 264.7 cells. Given its potent analgesic and anti-inflammatory activities in MIA rats and acetic acid-induced writhing in mice, HT083 should be further studied in order to explain its mechanism of actions in alleviating OA pain and inflammation.

## 1. Introduction

Osteoarthritis (OA) is the most prevalent progressive joint disease among the elderly population worldwide characterized by the erosion of cartilage, synovial inflammation, subchondral bone alteration, which causes pain, stiffness, and the loss of movement [1]. According to a new study, nearly half the total population aged over 65 or older throughout the world have OA symptoms, and 25% of them are suffering from severe disabling pain [2]. Chronic OA pain is linked to the erosion of joint cartilage caused by inflammation, and when it gets worse, bones can come into contact with each other causing severe pain and even the loss of movement [3]. As synovial inflammation is one of the major signs of OA, the role of inflammatory cytokines and mediators in the progression of OA is now well recognized. Synovium and chondrocytes produce and release cytokines and inflammatory mediators in the synovial fluid of OA patients [4]. As OA patients are mostly concerned with pain and inflammation, the current therapeutic strategies are designed exclusively to suppress pain and inflammation. Although non-steroidal anti-inflammatory drugs (NSAIDS) and corticosteroids are currently being used for OA pain management, their long-term use in aged people is often associated with negative health consequences, including cardiovascular, renal, and gastro-intestinal disorders [5,6]. The demand for an alternative OA treatment which is safe and effective is therefore increasing.

Bioactive natural products are drawing tremendous attention as alternative treatment for many health problems thanks to their high level of safety and efficacy [7]. Extensive research studies have been conducted with numerous traditional medicinal herbs to develop alternative treatments for inflammatory diseases including arthritis [8]. We selected *Paeonia lactiflora* Pall root and *Commmiphora myrrha* gum resin for their potential antiarthritic properties. *P. lactiflora* is a traditional medicine which has been used to treat pain, inflammation, and immune disorders for more than 1000 years in China [9]. The root of *P. lactiflora* contains several pharmaceutically active constituents, such as paeoniflorin, albiflorin, and penta-O-galloyl-β-d-glucose and is known to have anti-oxidant, anti-inflammatory, anti-viral, and anti-tumor activities [10]. *C. myrrha* is native to Northeastern Africa; its aromatic gum resin has been widely used for the treatment of rheumatoid arthritis, sinusitis and cough, gum and gingival problems, and sore throat [11]. Diverse secondary metabolites including terpenoids, steroids, flavonoids, sugars, lignans, etc. have been discovered in this genus [12]. Both *P. lactiflora* root and *C. myrrha* demonstrated excellent antinociceptive and anti-inflammatory properties in animal models, as well as cartilage protection and anti-inflammatory properties in in vitro assays from our preliminary experiments.

The use of herbal formulae in ancient as well as in modern natural medicines is well-documented. Historically, herbal formulas are regarded to be superior to individual herbal treatments in terms of potency and safety, as the individual herbs can produce synergistic effects and can neutralize any potential toxicity through interactions [13]. Although *P. lactiflora* and *C. myrrha* have been used in traditional medicines for treating joint diseases, no studies have investigated the mixture of these two herbs against OA. For our study, *P. lactiflora* and *C. myrrha* were mixed in a 3:1 (w/w) ratio, chosen after a number of trials. The Commiphora extract mixture was termed as HT083.

Monosodium iodoacetate (MIA)-induced animal model of OA is well-known for reproducing the symptoms as in the case of OA patients [14]. When injected into the joint cavity, MIA disrupts the glycolytic energy metabolism in chondrocyte and causes cell death, leading to inflammation and cartilage damage [15]. Acetic acid-induced writhing is a well-established model to assess the peripheral analgesic activities of natural and conventional medicines, which can quantitatively measure pain [16]. Acetic acid can induce pain by releasing prostaglandin, histamine, serotonin, and cytokines in the peritoneal fluid [16]. Lipopolysaccharide (LPS)-induced RAW 264.7 cells are a widely accepted in vitro model to study inflammatory responses, which release various inflammatory cytokines, chemokines, and mediators, such as nitric oxide (NO), inducible nitric oxide synthase (iNOS), cyclooxygenase-2 (COX-2), tumor necrosis factor-α (TNF-α), interleukin-1β (IL-1β), and interleukin-6 (IL-6) and so on *via* the activation of the inflammatory pathways, such as nuclear factor-kB (NF-kB) and mitogen-activated protein kinases (MAPKs) [17]. The present study aims to evaluate the aminociceptive and the anti-inflammatory activities of HT083 against OA using MIA model of rat, acetic acid-induced mouse model of writhing, and LPS-stimulated RAW 264.7 macrophages.

## 2. Results

### 2.1. HPLC Analysis of P. lactiflora and C. myrrha Extracts

**Figure 1:**
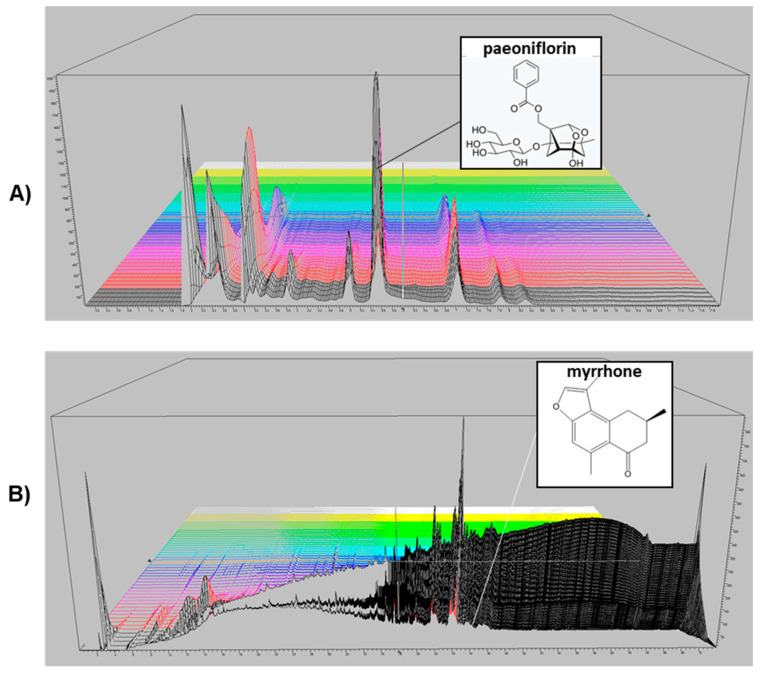
3-D HPLC chromatogram of *Paeonia lactiflora* and *Commiphora myrrha* extracts. Detection was performed by using a photodiode array detector. X-axis is retention time; Y-axis is wavelength, and Z-axis is absorbance unit. Analytical conditions were as follows: column, C18 (4.6 × 250, 5 µm); mobile phase, solvent A (0.1% phosphoric acid) and solvent B (acetonitrile); flow rate, 1 mL/min; program, 20–20%; 15–15.1 min, 20–70%; 15.1–20 min, 70–70%; 20–20.1 min, 70–20%; 20.1–25 min, 20–20% solvent B for *P. lactiflora* extract (**A**). Additionally, 0–60 min, 0–100%; 60–65 min, 100–100%; 65–67 min, 100–0%; 67–72 min, 0–0% solvent B for *C. myrrha* extract (**B**).

The main compounds in *P. lactiflora* and *C. myrrha* extracts were identified as paeoeniflorin and myrrhone, respectively using HPLC-UV method. A three-dimensional HPLC chromatogram including the structure of the constituent compounds is shown in Figure 1A,B. The paeoniflorin content in *P. lactiflora* was 79.9 mg/g (8%) and the myrrhone content in *C. myrrha* was 0.54 mg/g (0.05%).

### 2.2. Weight-Bearing Distribution in Monosodium Iodoacetate (MIA) Rats

**Figure 2:**
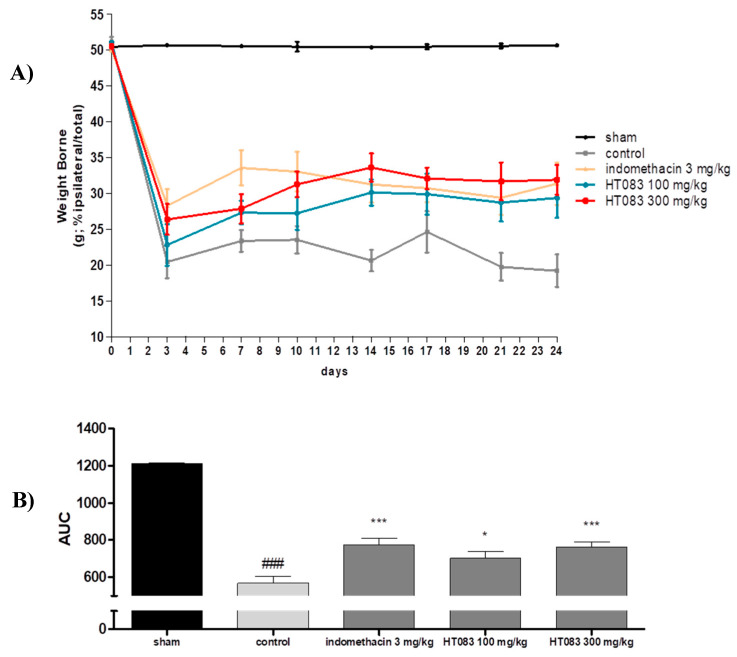
Effects of HT083 on the change of weight-bearing in rats injected with monoiodoacetate (MIA). (**A**) Distribution ratio of right and left hind-limb weight-bearing of the MIA rats after 24 days of MIA injection. The positive control (PC) was indomethacin (10 mg/kg). (**B**) AUC, area under the curve; evaluated the right and left hind-limb weight-bearing in MIA rats after 24 days of MIA injection. The values were expressed as the mean ± S.E.M. (*n =* 9). ### *p* < 0.001 vs sham, * *p* < 0.05, and *** *p* < 0.001 vs control.

The changes in weight-bearing distribution between the right and left hind limbs is an indicator of joint discomfort and pain, and this index is frequently used in rodent models to evaluate the antinociceptive activities of natural products and compounds against OA [18]. The weight-bearing distribution between the right and the left limbs measured during the treatment period for 24 days after MIA injection is shown in Figure 2A. The MIA injection triggered a dramatic loss in the weight-bearing capacity in the rats since day 3 and continued to be significantly lower compared to the sham group throughout the period. However, the supplementation of HT083 to the MIA rats resulted in a considerable improvement in the weight-bearing. Noticeably, 300 HT083 had a similar level of effect with the indomethacin in increasing weight-bearing capacities in the rats (Figure 2B).

### 2.3. Knee Joints and Joint Cartilage by HT083

**Figure 3:**
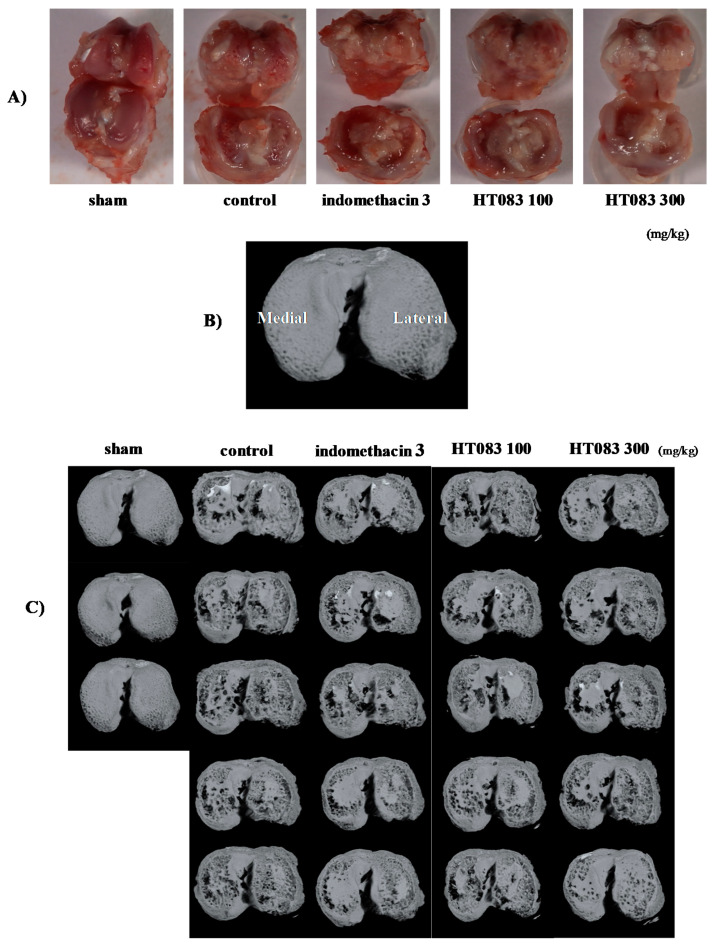

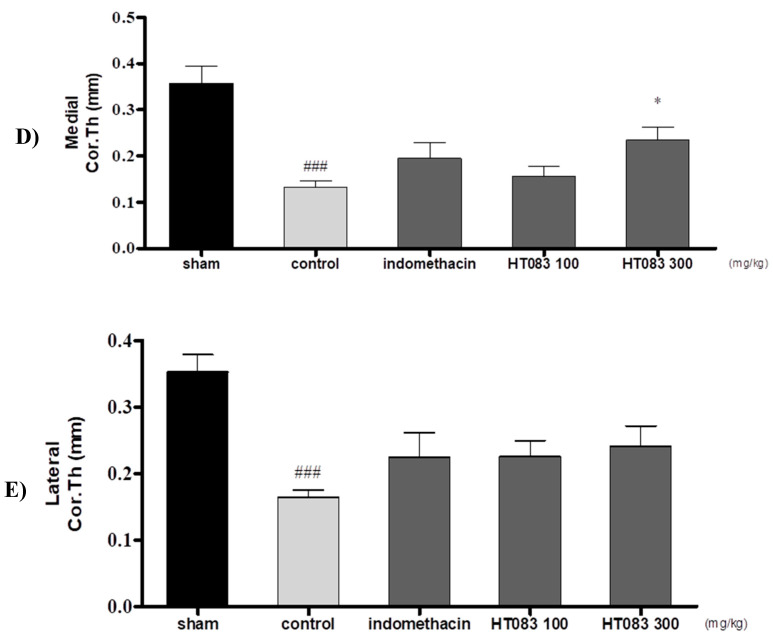
Effects of HT083 on the cartilage and subchondral bone damage. (**A**) Macroscopic images of the articular surfaces of tibia and femur of the sham and MIA rats (**B**) Axial Micro-computed tomography (Micro-CT) image of a rat tibia, in the medial (M) and lateral (L) subchondral bone compartment (**C**) Micro-CT images of the cartilage surface of the sham and MIA rats. (**D**,**E**) Histomorphometry parameters obtained by micro-CT of subchondral medial and lateral cortical thickness (Cor.Th). Each value is the mean ± SEM. The number of animals was nine in each group; * *p* < 0.001 vs. control and ^###^ *p* < 0.001 vs sham (one-way ANOVA, Dunnett’s post hoc test).

The photographic images of the knee joints of the rats show that MIA inflicted enormous cartilage damage, which was improved by HT083. As shown in Figure 3A, the sham rat had a normal and smooth cartilage in the knee joint, whereas the MIA-injected control rat had a damaged cartilage with a rougher and less polished surface. The HT083 recovered the MIA-inflicted cartilage loss, and the cartilage recovery in the 300 HT083-fed rat was higher than the indomethacin-treated rat. The MIA-injected rats also had a huge loss of subchondral bone in the central, medial, and lateral tibia, while this bone erosion was mitigated with 300 HT083 treatment as observed in the micro-CT images (Figure 3C). The micro-CT analysis of the medial and lateral subchondral tibia show that MIA caused a cortical bone thinning, and 300 HT083 supplementation resulted in a noticeable improvement in the cortical bone (Figure 3D,E).

### 2.4. Serum IL-1β in MIA-Induced Osteoarthritis (OA) Rats

**Figure 4:**
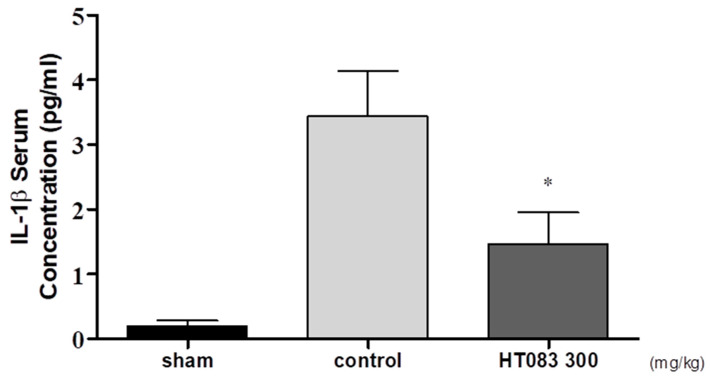
The change of serum IL-1β concentration in MIA rats after HT083 treatment. Each value is the mean ± SEM. The number of animals is nine per group; * *p* < 0.05, * *p* < 0.01 and * *p* < 0.001 vs. control (one-way ANOVA, Dunnett’s post hoc test).

The presence of pro-inflammatory cytokines increases in the serum of OA subjects as the disease progresses [19]. HT083 has shown a potent anti-inflammatory effect by suppressing the serum level of IL-1β, a key modulator in OA inflammation. As shown in Figure 4, there was a dramatic increase in the concentration of IL-1β in the control group injected with only MIA, whereas HT083 (300 mg/kg) treatment attenuated the serum IL-1β level of the MIA rats (Figure 4).

### 2.5. Effects of HT083 on Acetic Acid-Induced Writhing in Mice

**Figure 5:**
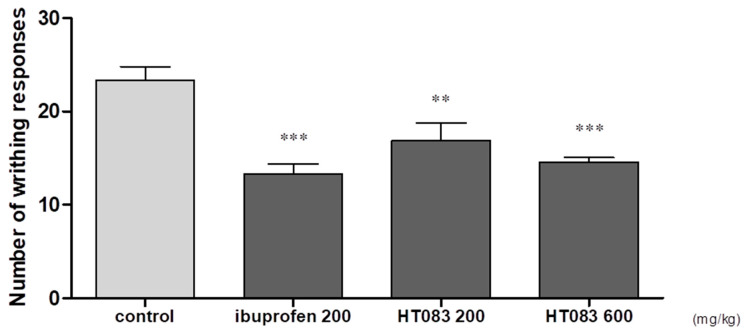
Effects of HT083 on acetic acid-induced writhing response in rat. The number of writhes was measured after 0.7% acetic acid injection for 10 min. Each value is the mean ± SEM. The number of animals is eight per group; ** *p* < 0.01 and *** *p* < 0.001 vs. control (one-way ANOVA, Dunnett’s post hoc test).

There is a growing consensus that acetic acid-induced writhing in laboratory animals simulates the peripheral pain originating from inflammatory and other diseases. Acetic acid is known to induce pain by producing pain mediators, including prostaglandin, and the pain can be measured quantitatively by observing the writhing responses [20]. In the current study, acetic acid was orally administered, and the writhing responses of the mice were recorded over 10 min. It was found that the acetic acid-induced writhing count was reduced by HT083 treatments. The reduction of writhing responses by HT083 at 600 mg/kg was 37%, which was comparable to that of the standard drug, ibuprofen (40%) at 200 mg/kg (Figure 5).

### 2.6. Effects of HT083 Against Inflammatory Responses in Lipopolysaccharide (LPS)-Stimulated RAW264.7 Cells

**Figure 6:**
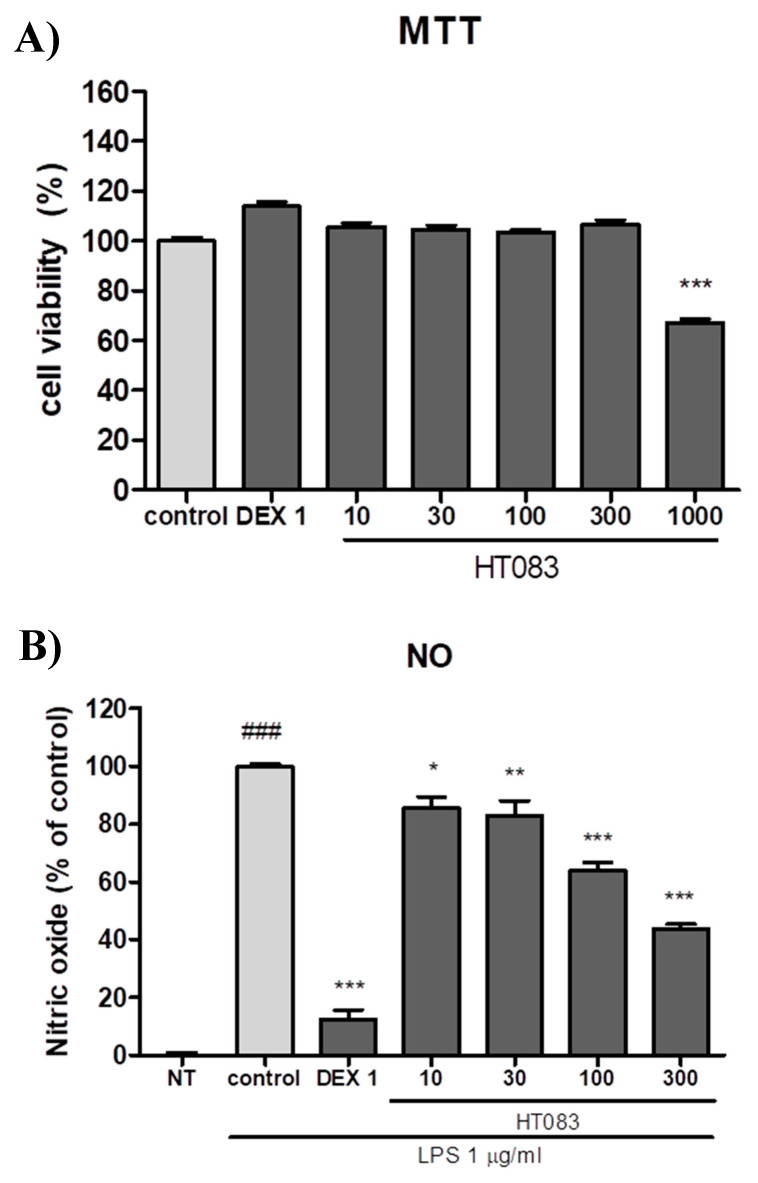
Effects of HT083 on cell viability (**A**) and NO production (**B**) in lipopolysaccharide (LPS)-stimulated RAW 264.7 cells. Cells were pretreated with 10, 30, 100, and 300 μg /mL of HT083. Each value is the mean ± SEM. * *p* < 0.05, ** *p* < 0.01 and *** *p* < 0.001 vs. control; ^###^ *p* < 0.001 vs. no treatment (NT) (one-way ANOVA, Dunnett’s post hoc test). MTT; thiazolyl blue tetrazolium bromide, NO; nitric oxide.

The anti-inflammatory activities of HT083 were evaluated in LPS-induced RAW264.7 macrophages. HT083 showed potent anti-inflammatory effects by lowering the NO concentration and the expression of IL-1β, IL-6, iNOS, and COX-2. No potential cytotoxicity of HT083 was observed with up to 300 µg/mL in the RAW264.7 cells (Figure 6A). The LPS- stimulated NO production was decreased by HT083 in a dose-dependent manner. Noticeably, ~50% NO reduction was obtained with 300 HT083 compared to the control (Figure 6B). The anti-inflammatory effects of HT083 in LPS-induced RAW264.7 cells were assayed through Quantitative Real Time-PCR (qRT-PCR), ELISA, and western blotting.

**Figure 7:**
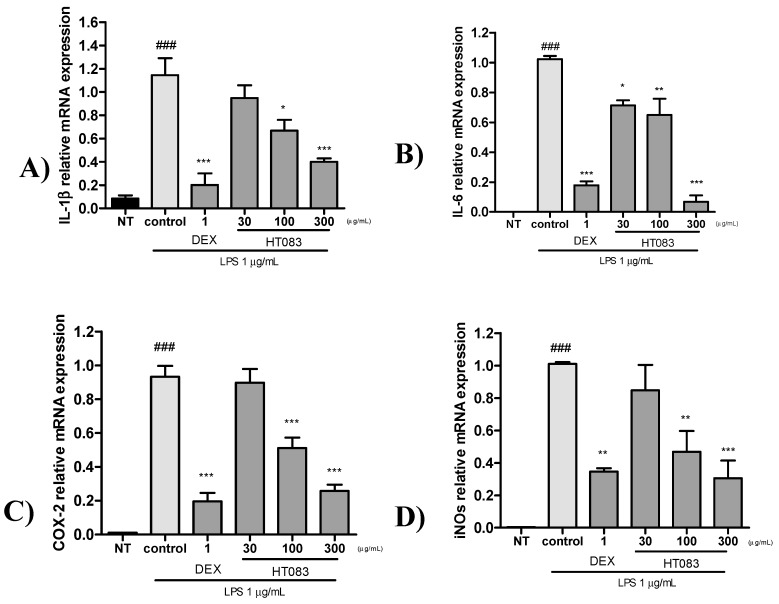
Quantitative Real Time-PCR (qRT-PCR) analysis of IL-1β, IL-6, COX-2, and iNOS mRNA expression in LPS-stimulated RAW264.7 cells. Cells were pretreated with positive control, Dexmethason 1 μg/mL and HT083 (30, 100, 300 μg/mL), and then incubated with LPS for 24 h. The cells were lysed, and total RNA was subjected to quantitative Real Time-PCR with the primers IL-1β, IL-6, COX-2 and iNOS (**A**–**D**). Each experiment was repeated three times. Each value is the mean ± SEM. The number of animals is nine per group; * *p* < 0.05, ** *p* < 0.01 and *** *p* < 0.001 vs. control; ^###^ *p* < 0.001 no treatment (NT) (one-way ANOVA, Dunnett’s post hoc test).

As shown in Figure 7A, the mRNA expression of IL-1β, IL-6, iNOS, and COX-2 dramatically increased in LPS-stimulated cells. All four cytokines in the cells were suppressed by both HT083 (30–300 µg/mL) and dexmethason (1 µg/mL). The anti-inflammatory effect of HT083 was dose-dependent, and 300 HT083 showed similar level of effects with dexmethason.

**Figure 8:**
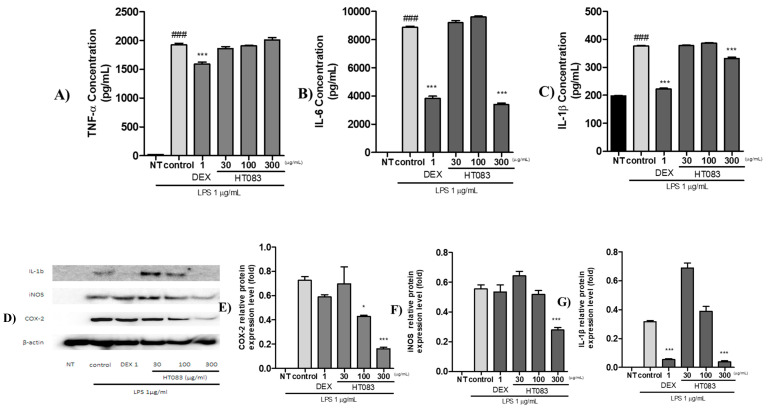
Effects of HT083 on the protein expression of inflammatory cytokines in LPS-stimulated RAW264.7 cells. (**A**–**C**) Multiplex analysis of the expression of TNFα, IL-6, and IL-1β. Cells were stimulated with PBS, trophozoite lysates or LPS for 24 h. Supernatants were harvested and inflammatory cytokine concentrations (pg/mL) were measured. * *p* < 0.05, *** *p* < 0.001 indicate statistically significant differences from the control group. ^###^ *p* < 0.001 indicates significant difference from the non-treated (NT) group. (**D**–**G**) Western blot image and analysis of the expression of COX-2, iNOS and IL-1β.

As illustrated in Figure 8, the protein expression of the pro-inflammatory cytokines and mediators, such as IL-6, and IL-1β, iNOS, and COX-2 were suppressed by HT083 treatment in LPS-stimulated RAW264.7 cells. 300 HT083 lowered the concentrations of IL-6 and IL-1β in RAW264.7 cells to the level of the positive control (Figure 8A–C). As shown in the western blot image, the expression of IL-1β, iNOS, and COX-2 were dose-dependently lowered by HT083 (Figure 8D). Remarkably, 300 HT083 showed higher anti-inflammatory effects against all three cytokines than the positive control.

## 3. Discussion

The herbal mixture of *P. lactiflora* root and *C. myrrha* gum HT083 in this study has shown remarkable analgesic properties by increasing weight-bearing capacities in MIA rats and decreasing writhing responses in mice induced with acetic acid. Additionally, HT083 suppressed inflammation by down-regulating pro-inflammatory cytokines and mediators in MIA rats and in RAW264.7 cells stimulated with LPS. Furthermore, the cartilage degeneration and subchondral bone erosion in MIA rats were recovered with HT083 administration.

The change in weight-bearing capacity measured by a behavioral test, known as incapacitance test, is widely accepted as an outcome of painful behavior in MIA rats given that the hind limbs of the rodent support less weight as the animal feels pain [21]. MIA is a glycolytic inhibitor which inhibits the glycolysis of condrocytes that leads to the cell death and triggers inflammation, resulting in the cartilage degeneration and subchondral bone alteration in the knee joint [22]. The intraperitoneal administration of MIA into rats is a widely accepted and an archetypical model of studying OA, as it can display much of the nociceptive behaviors including the pathological attributes, such as synovial tissue inflammation and cartilage degradation [23]. The incapacitance test was conducted in MIA-induced OA rats for 24 days, and it was observed that the weight-bearing capacity of the MIA-injected rats was highly improved with HT083 treatment. Although indomethacin was found more effective in increasing weight-bearing in the initial days of the disease following the MIA injection, HT083 has shown superior efficacies than indomethacin after day 11. While the early stage of MIA-induced experimental OA is characterized by macrophage infiltration and synovial inflammation; the later stage of MIA-induced OA is associated with severe cartilage loss and subchondral bone remodeling [22,24]. Presumably, HT083 has provided greater protection against cartilage erosion and higher weight-bearing capacity than indomethacin to the MIA rats in the late stage of the disease. In addition, it is known that the central analgesics like spinal cannabinoids can perform better to relieve pain than NSAIDs at the advanced stage of OA [25]. It has been suggested that *C. myrrha* exerted analgesic functions via activating the brain opioid mechanisms in mice [26]. A recent study reported that paeoniflorin, obtained from *P. lactiflora* can mitigate inflammatory pain by suppressing Akt-NF-kB pathway [27]. Our study has identified paeoniflorin in *P. lactiflora* as a major compound (8%) from HPLC analysis. It can be suggested that the central analgesic effects of *C. myrrha* and the inhibitory effects *P. lactiflora* against inflammatory pain might have contributed to the pain relief and increase in weight-bearing at the late stage in MIA rats. The increased weight-bearing capacity of the hind limbs by HT083 in the rats indicates its antinociceptive effect in the arthritic knee [28]. Since OA pain induces the secretion of substance P of nerve fibers and further aggravates the inflammation, suppressing the pain not only improves the quality of life but also inhibits further progression of arthritis. As both peripheral and central mechanisms are associated with arthritic pain in MIA model, presumably HT083 has suppressed pain of both peripheral and central origin [29]. *C. myrrha* and *P. lactiflora* have been historically used as natural antinociceptives since ancient times, and both have proven functions against peripheral and central pains [30,31]. This study demonstrated the combined therapeutic effects as well as the synergistic effects of *C. myrrha* and *P. lactiflora* to treat OA pain and inflammation in MIA rats. According to literature, the pathological characteristics of joints and cartilage observed in OA induced with MIA resemble those of human OA [32]. Therefore, the pain relief in MIA-induced OA rats by HT083 in the present study implies a similar effect in human OA.

Pro-inflammatory cytokines play a significant role in the pathophysiology of OA by accelerating synovial inflammation as well as cartilage destruction [33]. In particular, IL-1β is considered a key mediator in the progression of OA inflammation [34]. It has been suggested that IL-1β initiates an initial response to OA inflammation by up-regulating the generation of extracellular protein breakdown enzymes that damage cartilage cells in OA, while down-regulating the biosynthesis of proteoglycan and collagen, the main components of cartilage [35]. This study has found that IL-1β in serum was increased after MIA injection in rats, whereas HT083 significantly reduce the serum IL-1β to the level equivalent to the positive control. The excessive productions of IL-1β along with other pro-inflammatory cytokines promote OA progression by inducing inflammation and cartilage damage leading to the decrease in weight-bearing capacity [36] Therefore, the decrease of serum IL-1β by HT083implicates the suppression of the initial response to inflammation and the improvement in weight-bearing capacities of the rats.

The degeneration of cartilage and the erosion of subchondral bone are two important pathological symptoms of OA [37]. The photographic images of the cross-section and the micro CT images of the knee joints of the MIA rats show that the suppression of pain by HT083 was co-occurred with the improvement of the knee joint structure. A noticeable recovery of the cartilage structure was observed in the MIA rats fed with HT083 compared to the non-treated rats. Research evidence indicates that MIA triggers the disruption of cartilage matrix and impairs joint structure in experimental animals resulting in the behavioral and pathophysiological characteristics identical to human OA [38]. A marked recovery of the MIA-inflicted subchondral bone loss was recovered with HT083 treatment as seen in the micro CT images. Literature suggests that the degeneration of cartilage and the remodeling of subchondral bone architecture in arthritic knees are occurred by the inflammatory responses in the synovial fluid [39]. According to a previous study, cortical bone density was decreased in the MIA rats as compared with the normal rats [40]. The report indicated that the subchondral microarchitecture could be modified due to remodeling occurred by MIA administration. Similarly, our study found that the cortex of the medial and lateral tibia of the MIA rats was damaged, which was substantially recovered with HT083 supplementation. In summary, HT083 provided a remarkable protection against MIA-induced cartilage and subchondral bone damage.

Even though the role of central inputs in OA pain is well recognized, clinical research evidence emphasizes that OA pain is occurred predominantly by peripheral inputs, as joint replacement or the treatment with peripheral analgesics can alleviate OA pain in most of the cases [41]. Acetic acid-induced writhing test was performed to assess the antinociceptive effects of HT083 against peripheral pain. In acetic acid-induced writhing, the activities of an analgesic agent are quantitatively measured from the changes of writhing responses [16]. HT083 in this study dose-dependently decreased the acetic acid-induced writhing in mice. After being injected, acetic acid induces pain by releasing pain mediators, which leads to the writhing response in the animal [42]. By decreasing the acetic acid-induced writhing response, HT083 has shown a high efficacy against peripheral pain.

RAW264.7 cells are widely used as an in vitro inflammatory model to study the effectiveness of anti-inflammatory agents [43]. When stimulated with LPS, these immune cells release inflammatory cytokines as an inflammatory response. The current study has shown that HT083 can effectively suppress the LPS-induced inflammatory responses in RAW26.7 cells. HT083 remarkably suppressed the LPS-induced overproduction of NO and the inflammatory cytokines, such as IL-1β, IL-6, iNOS, and COX-2. Reports indicate that the increased amounts of COX-2 and iNOS in the synovial fluid triggers the generation of several pro-inflammatory cytokines, chemokines, and mediators causing the destruction of cartilage structure and synovial swelling, which leads to joint pain and stiffness [44,45]. Therefore, the efficacy of HT083 against the inflammatory responses in RAW264.7 cells as well as in MIA rats indicates that HT083 could help relieve OA inflammation.

This study is the first to show that the herbal formula of *P. lactiflora* and *C. myrrha* (HT083) has analgesic and anti-inflammatory effects in MIA-induced OA in rats and acetic acid-induced writhing in mice. Importantly, HT083 has been more efficacious than the standard drug in decreasing weight-bearing in MIA rats. The prevention of cartilage degeneration and subchondral bone damage in the affected knees as well as the suppression of the inflammatory cytokines and mediators in MIA rats and in LPS-activated RAW264.7 macrophages by HT083 was also remarkable. In addition, the acetic acid-induced writhing in mice, which quantitatively measures pain, was substantially reduced by HT083. Further extensive studies could explain the molecular mechanisms of the analgesic and the anti-inflammatory effects of this new herbal formula.

## 4. Materials and Methods

### 4.1. Sample Preparation and HPLC Analysis

The dried root of *P. lactiflora* and the dried gum resin of *C. myrrha* used in the experiment were obtained from Daewoo-pharmacist *Co. LTD.* (Seoul, Korea) and Hyun-Jin pharmaceutical Co. LTD. (Seoul, Korea), respectively. The materials were identified by Professor Dong-Hun Lee, Department of Herbal medicine, College of Oriental Medicine, Gachon University, where the voucher specimens (18101802 and 18110104, 18110107) are preserved. Dried *P. lactiflora* and *C. myrrha* were extracted with 30% EtOH under reflux at 80 ℃ for 3 h. Then the extract was filtered and the ethanol was removed under decompressed condition before lyophilizing the extract at −80 ℃. A mixture of *P. lactiflora* and *C. myrrha* (HT083) with a ratio of 3:1 (w/w) was prepared for the experiment. The chromatographic analysis of each sample was performed with 1260 Infinity Ⅱ HPLC system (Agilent, USA). The separation was done with an Eclipse XDB C18 column (4.6 × 250 mm, 5 µm, Agilent, USA) at 35 ℃. The sample (100 mg) was sonicated in 10 mL of 50% methanol for 10 minutes and filtered using a 0.45 μm syringe filter (Waters Corp., Milford, USA). The mobile phase was composed of 0.1% phosphoric acid (A) and acetonitrile (B). The injection volume was 10 μL and the flow rate was 1.0 mL/min. The effluent was monitored at 235 and 254 nm. Each extract was analyzed in triplicate.

### 4.2. Animal Management, MIA Injection, and Diet Preparation

Male Sprague-Dawley (SD) rats (5-week-old) and ICR mice (6-week-old male) were purchased from Samtako Inc. (Osan, Korea). The animals were maintained in a room under standard laboratory condition with a 12:12 h light-dark cycle with a constant temperature of 22 ± 2 ℃ and humidity of 55 ± 10%. The animals were acclimatized for a week before the experiment. Rats had access to ad libitum feed and drink. The experiment was conducted in compliance with the current ethical regulations for animal care and use at Neumed Inc., (Seoul, Korea) (KISTEM-IACUC-2019-001 at 22/02/2019 and KISTEM-IACUC-2018-002 at 27/04/2018).

SD rats were randomly divided into five groups: sham, control, indomethacin, 100 HT083, and 300 HT083. Rats were first anesthetized using 2% isofluorane O_2_ mixture and then 50 μL of 40 mg/mL of MIA (Sigma, USA) dissolved in 0.9% saline (Sigma, USA) was intra-articularly injected into the knee joint using a 26 gauge 0.5-inch needle. The experimental groups were treated as follows: sham and control groups were fed only basic diet (AIN-39G), indomethacin group fed AIN-93G diet containing 0.003% indomethacin so that the final dose could be 3 mg/kg, 100 HT083 group fed AIN-93G diet containing 0.11% HT083 so that the final dose could be 100 mg/kg, and 300 HT083 group fed AIN-93G diet containing 0.33% HT083 so that the final dose could be 300 mg/kg. From the day of MIA injection, the diet was served for 24 days at 18 g per 200 g body weight per day.

### 4.3. Weight-Bearing Capacity

Following the MIA injection, hind limb weight-bearing was measured on 0, 3rd, 7th, 10th, 14th, 17th, 21st, and 24th days using an Incapacitance Meter Tester 600 (IITC Life Science Inc., Woodland Hills, USA) for 10 s. The weight-bearing capacity of the right hind limb was calculated using the below formula: weight bearing ratio (%) = (weight on right hind limb / weight on left hind limb + weight on right hind limb) × 100.

### 4.4. Serum IL-1β Analysis of MIA Rats

The whole blood was taken from the abdominal vein. The samples were centrifuged for 10 min at 4000 rpm, and the isolated serum was preserved at −70 °C until use. Multiplex assay was performed using IL-1β Rat Premixed Multi—Analyte Kit (R&D Systems Inc., Minneapolis, USA) for serum IL-1β measurement following the manufacturer’s procotol. The data were analyzed with a Luminex MAGPIX analyzer (Luminex Co., Austin, USA).

### 4.5. Micro-Computed Tomography (Micro-CT) Analysis

The right hind limbs of the animals were removed and fixed with formaldehyde (Sigma, USA). The samples were scanned with SKYscan 1176 micro-CT system (Skyscan, Kartuizersweg, Belgium). The 2D and 3D imaging and analysis were performed with NRecon software (Skyscan v. 1.6.10.1), DataViewer software (Skyscan v.1.5.1.9), CTAn software (Skyscan v.1.17.7.2), CTvol software (Skyscan v. 2.3.2.0) with following conditions: X-ray source: 70 kV/355 µA, Exposure time: 800 ms, Filter: 0.5 mm aluminum, Pixel size 8.9 µm, Rotation angle: 180° rotation angle with rotation steps of 0.4°.

### 4.6. Measurement of Acetic Acid-Induced Writhing Response

ICR mice were randomly divided into four groups. Acetic acid (0.7%) was intraperitoneally injected into the mouse at 10 mL/kg body weight. After 10 min of the injection, the writhing responses (abdominal constrictions) of the mouse were recorded for 30 min inside an observation chamber. The mice were pretreated with the vehicle, ibuprofen (200 mg/kg), and HT083 (300 and 600 mg/kg) 30 min before the MIA injection. Significant reduction in the writhing responses in the experimental groups compared to the control was regarded as analgesic response.

### 4.7. Cell Culture

RAW 264.7 cells were obtained from Korean Cell Line Bank (Seoul, Korea). The cells were maintained at 37 °C and 5% CO_2_ with DMEM medium containing 10% FBS, 100 IU/mL penicillin, and 100 μg/mL streptomycin (Gibco BRL, Grand Island, NY, USA).

### 4.8. NO Generation and Cell Toxicity Measurement

RAW 264.7 cells were cultured at 37 °C and 5% CO_2_ for 24 h. After seeding, cells were incubated with different concentrations of HT083 (10–300 µg/mL) and LPS (1 µg/mL) for 24 h. Greiss reagent was added to the culture supernatant (1:1) and the absorbance was measured at 540 nm. Cytoxicity was measured using the MTT assay. Briefly, 5 mg/mL of MTT reagent was added to RAW 264.7 cells and incubated at 37 °C and 5% CO_2_ for 1 h. After removing the supernatant, 100 μL of DMSO was added and kept for 10 min before measuring the absorbance at 540 nm.

### 4.9. Quantitative Real-Time Polymerase Chain Reaction (qRT-PCR) Analysis

Total RNA was extracted using QIAzol Lysis reagent (Qiagen Ltd., Manchester, UK). cDNA was generated using a Reverse Transcription Kit (R&D Systems, USA) following the manufacturer’s protocol. Quantitative real-time PCR (qRT-PCR) was performed with StepOnePlus real-time PCR system (Applied Biosystems, CA, USA). cDNA was amplified using Power SYBR^®^ Green PCR Master mix (Applied Biosystems, CA, USA). The primer sequences used in the experiment are indicated in Table 1. 

### 4.10. Protein Expression Analysis

RAW 264.7 cells were treated with 30, 100, and 300 µg/mL of HT083 and 1 µg/mL of LPS for 24 h. Total protein was extracted with PRO-PREP^TM^ Protein Extraction Solution (iNtRON Biotech, Seongnam, Korea). Equal amounts of protein (µg) were separated by sodium dodecyl sulfate-polyacrylamide gel electrophoresis (SDS-PAGE) and transferred to nitrocellulose membranes for 2 h at 100 mA constant current/cm^2^ followed by blocking for 1 h. The blots were then incubated overnight with the primary antibodies (iNOS, COX-2, and IL-1β) and probed with the secondary antibody at room temperature for 2 h. The blots were visualized with an Amersham Imager^TM^ 600 (GE Healthcare Bio-sciences Corp., Piscataway, USA) using Pierce™ ECL Western Blotting Substrate (Thermo Scientific, Waltham, USA) solution. The multiplex assays were performed using Mouse Premixed Multi - Analyte Kit (R&D Systems, USA) and the results were measured and analyzed with Luminex MAGPIX analyzer (Luminex Corp., TX, USA) following the manufacturer’s protocols.

### 4.11. Statistical Analysis

All statistical analyses were performed with GraphPad Prism^®^ 5.0 (GraphPad Software, CA, USA) using One-way ANOVA and Dunnett’s post hoc test. The significance was determined at *p* < 0.05, *p* < 0.01, and *p* < 0.001. All experimental data were expressed as mean ± standard error of the mean.

## Figures and Tables

**Table 1:** mRNA primer sequence for Quantitative Real-Time RT-PCR analysis.

**IL-6**	F	5′-ACCAGAGGAAATTTTCAATAGG-3′
	R	5′-TGATGCACTTGCAGAAAACA-3′
**COX-2**	F	5′-AACCGCATTGCCTCTGAAT-3′
	R	5′-CATGTTCCAGGAGGATGGAG-3′
**IL-1β**	F	5′-CCTAAAGTATGGGCTGGACTGT-3′
	R	5′-GACTAAGGAGTCCCCTGGAGAT-3′
**iNOS**	F	5′-CCCTTCCGAAGTTTCTGGCAGCAGC-3′
	R	5′-GGCTGTCAGAGCCTCGTGGCTTTGG-3′
**GAPDH**	F	5′-TGGCCTCCAAGGAGTAAGAAAC-3′
	R	5′-CAGCAACTGAGGGCCTCTCT-3′

Interleukin-6 (IL-6); cyclooxygenase-2 (COX-2); Interleukin-1β (IL-1β); Inducible nitric oxide synthase (iNOS); Gliceraldehid 3-fosfat dehidrogenaza (GAPDH).
